# Multiallelic models for QTL mapping in diverse polyploid populations

**DOI:** 10.1186/s12859-022-04607-z

**Published:** 2022-02-14

**Authors:** Alejandro Thérèse Navarro, Giorgio Tumino, Roeland E. Voorrips, Paul Arens, Marinus J. M. Smulders, Eric van de Weg, Chris Maliepaard

**Affiliations:** Plant Sciences Group, Department of Plant Sciences, Wageningen University and Research, Droevendaalsesteeg 1, P.O. Box 386, 6700 AJ Wageningen, The Netherlands

**Keywords:** Polyploidy, Multiparental, QTL, Multiallelic, Genetic diversity

## Abstract

**Abstract:**

Quantitative trait locus (QTL) analysis allows to identify regions responsible for a trait and to associate alleles with their effect on phenotypes. When using biallelic markers to find these QTL regions, two alleles per QTL are modelled. This assumption might be close to reality in specific biparental crosses but is unrealistic in situations where broader genetic diversity is studied. Diversity panels used in genome-wide association studies or multi-parental populations can easily harbour multiple QTL alleles at each locus, more so in the case of polyploids that carry more than two alleles per individual. In such situations a multiallelic model would be closer to reality, allowing for different genetic effects for each potential allele in the population. To obtain such multiallelic markers we propose the usage of haplotypes, concatenations of nearby SNPs. We developed “mpQTL” an R package that can perform a QTL analysis at any ploidy level under biallelic and multiallelic models, depending on the marker type given. We tested the effect of genetic diversity on the power and accuracy difference between bi-allelic and multiallelic models using a set of simulated multiparental autotetraploid, outbreeding populations. Multiallelic models had higher detection power and were more precise than biallelic, SNP-based models, particularly when genetic diversity was higher. This confirms that moving to multi-allelic QTL models can lead to improved detection and characterization of QTLs.

**Key message:**

QTL detection in populations with more than two functional QTL alleles (which is likely in multiparental and/or polyploid populations) is more powerful when using multiallelic models, rather than biallelic models.

**Supplementary Information:**

The online version contains supplementary material available at 10.1186/s12859-022-04607-z.

## Introduction

Quantitative trait locus (QTL) analyses are those experiments in which a population is genotyped with many markers that cover the whole genome, and phenotyped for traits of interest. Once that is done, positions along the genome are tested for association, either defined by the markers or by some clever estimate such as those used in interval mapping [1, 2]. QTL studies have been extremely useful in unravelling genomic regions that control or contribute to important plant traits such as disease resistance, yield, crop quality or tolerance to abiotic stresses. The precision of these studies has been improved by the advent of high-throughput technologies, that facilitated genotyping of thousands to millions of Single Nucleotide Polymorphisms (SNPs) in a single analysis. This is nowadays also possible in polyploid organisms, thanks to statistical and computational developments in the areas of genotyping, linkage map construction and QTL analysis [3, 4].

When trying to find QTLs two aspects will define the outcome obtained: the type of population studied, and the QTL modelling approach chosen.

### Population types

A classical population type is the biparental cross, a population of siblings obtained by crossing two parents, usually contrasting in the trait of interest. If both parents are homozygous, as is the case in many self-fertilizing species, QTLs found in this type of population will reflect the allelic differences between the two parents. If the parents are diploids, there will likely be only two alleles per QTL segregating in that population. Since the cross contains only a small fraction of the genetic diversity of the species, QTL results from these populations may not be applicable to other populations and markers linked to QTLs cannot easily be used in other crosses.

Another possibility is to use a genome-wide association study (GWAS), in which a large set of diverse individuals are studied, and thus a large number of QTL alleles is expected to segregate. Unlike in biparental crosses, an association between markers and QTLs is expected due to Linkage Disequilibrium (LD) rather than direct family linkage. These studies produce more widely applicable QTL results, but introduce some drawbacks: (1) rare allele variants, which will be present at low frequency in a GWAS panel, will easily be missed even if they affect the phenotype, and (2) linkage disequilibrium (LD) is not spread homogeneously across the population or the genome, an effect known as “genetic structure”, and this may generate false positives if not taken into account [5, 6].

Nevertheless, as described in [7], mapping in biparental populations or GWAS panels represent two extremes of a genetic diversity gradient. An intermediate form can be found in multi-parental populations (MPP). An MPP is formed by individuals that share a limited number of known ancestors, for instance, a set of connected biparental crosses, or multiple lines originating from a small set of founders. As such, the number of QTL alleles will be at most of $ploidy \times founders$. Additionally, as the genetic structure in an MPP originates from mostly known pedigree relationships, it will be less complex than that of GWAS populations, and the allele frequencies will often be more balanced.

The MPP concept fits well the type of populations usually available in breeding programmes, where multiple crosses are made with some interesting parents. Breeding populations become then ad-hoc MPPs and instead of analysing each cross separately, the whole breeding program could be analysed at once, increasing statistical power. The idea that utilizing breeding populations for QTL analysis might be a better option than creating specific experimental populations has been studied previously [7–10], although in diploid species under biallelic models.

### Modelling approaches

The type of mathematical model used for QTL analysis will heavily depend on the population under study. In a classical biparental population an analysis of variance (ANOVA) will easily provide accurate QTL estimates. In contrast, in a GWAS panel, genetic structure must be taken into account, usually in the form of a mixed model [6]. In the case of a MPP, a similar mixed model could be used, although if the genetic structure is simple enough, a fixed factor accounting for subpopulations may perform well also [6].

The number of modelled QTL alleles is also relevant. Typically, since biallelic markers are used, two alleles per QTL are modelled. Assuming the presence of only two alleles, however, is sensible under very few scenarios. As ploidy, heterozygosity or the number of founders of a population increase, the number of expected QTL alleles rises. The larger the number of alleles, the less realistic the biallelic model becomes for describing the observed variance. Nevertheless, as SNP markers have become the standard polymorphism in modern genotyping, using them directly implicitly tests a biallelic scenario. However, SNP information can be used differently. By combining adjacent SNPs, biallelic SNPs can be turned into multiallelic haplotype markers [11].

Due to the increased genetic diversity present in GWAS and MPP populations, it is foreseeable that moving to multiallelic QTL models will provide a gain in statistical power. Nevertheless, biallelic models are simpler and thus more powerful, and they have a long trajectory of success. There is currently no software available that can perform multiallelic QTL analyses in polyploid populations in the presence of genetic structure, but such software is being developed. Under which circumstances, if any, will a genetically diverse population benefit from a multiallelic QTL modelling approach?

To answer this question, we have simulated a series of autotetraploid MPPs with different levels of genetic diversity. Populations were designed following the Nested Association Mapping (NAM) structure, where one central parent is crossed with many peripheral parents [12]. We adapted the QTL modelling approach presented in [13] for diploid MPPs with inbred founders, expanding it to a polyploid and heterozygous case. We present this approach as an R package [14] named “mpQTL” to perform QTL analysis. This package together with the simulated MPPs allowed us to assess the effect of biallelic or multiallelic markers on QTL detection and QTL precision under different genetic diversity scenarios.

## Materials and methods

### Statistical models

Mixed models allow to correct for dependence between observations due to genetic structure. Yu et al*.* (2006) defined a “unified mixed model”, also known as the $Q + K$ model [4], that can accommodate both a population structure matrix ($Q$) and a kinship matrix ($K$):

1 $$\begin{array}{*{20}c} {y = X{\varvec{\beta}} + Q{\varvec{v}} + \underline{{Z{\varvec{u}}}} + \user2{\underline {\varepsilon } }\quad Var\left( {\varvec{u}} \right) = K\sigma_{G}^{2} \quad Var\left( {\varvec{\varepsilon}} \right) = R\sigma_{\varepsilon }^{2} } \\ \end{array}$$

where $y$ is the vector of phenotypic trait values, $X{\varvec{\beta}}$ represents the incidence matrix and marker effects (SNP effect in [6]); $Q{\varvec{v}}$ are the population structure matrix and vector, respectively; $\underline{{Z{\varvec{u}}}}$ are design matrix and vector of genetic background effects (polygene component in [6]); and $\user2{\underline {\varepsilon } }$ is the residuals vector. The variances of the random effects, ${\varvec{u}}$ and ${\varvec{\varepsilon}}$ are also defined: $K$ is the kinship matrix and $\sigma_{G}^{2}$, the genetic variance; $R$ is a matrix with off-diagonal numbers being 0 and the diagonal is the reciprocal of the number of observations underlying each genotype estimation, and $\sigma_{\varepsilon }^{2}$ is the residual variance.

#### Fixed term: allele parametrization

Definition of $X$ requires a genetic model, that is, a method to transform genetic data into an incidence matrix $X$. Polyploid genetic models have existed for a long time [15] and have inspired more recent versions applied to SNP data [16, 17]. The simplest of them is the *biallelic model *(model B in [7], association mapping in [18]), which considers SNP alleles as equal to QTL alleles. In a biallelic model, the SNP dosages are used to predict genetic effects, giving the $X\beta$ term the following form:

2 $$\begin{array}{*{20}c} {X_{b} \beta = \left[ {\begin{array}{*{20}l} 1 \hfill & {\delta_{1} } \hfill \\ 1 \hfill & {\delta_{2} } \hfill \\ \vdots \hfill & \vdots \hfill \\ 1 \hfill & {\delta_{n} } \hfill \\ \end{array} } \right]\left[ {\begin{array}{*{20}c} \mu \\ \beta \\ \end{array} } \right] } \\ \end{array}$$

where $\delta_{i}$ are the dosages (a value from 0 to *ploidy*) of one of the SNP alleles, $\mu$ is the intercept and $\beta$ the genetic effect of that SNP allele. We denote the incidence matrix as $X_{b}$ for this modelling strategy. Note that this represents an additive model without intra or inter-locus interaction, i.e. no dominance or epistasis between alleles is modelled.

Alternatively, Identity-By-Descent (IBD) information can be used to generate an ancestral model [13], also known as a PBA model [10] or an LDLA model [9, 19]. Under the ancestral model, the dosage of each ancestral allele or haplotype in the NAM population is used to estimate genetic effects. The shape of the $X\beta$ term then takes the form:

3 $$\begin{array}{*{20}c} {X_{a} \beta = \left[ {\begin{array}{*{20}l} 1 \hfill & {\delta_{11} } \hfill & {\delta_{12} } \hfill & \hfill & {\delta_{1k} } \hfill \\ 1 \hfill & {\delta_{21} } \hfill & {\delta_{22} } \hfill & \hfill & {\delta_{2k} } \hfill \\ \vdots \hfill & \vdots \hfill & \vdots \hfill & { \ldots } \hfill & \vdots \hfill \\ 1 \hfill & {\delta_{n1} } \hfill & {\delta_{n2} } \hfill & \hfill & {\delta_{nk} } \hfill \\ \end{array} } \right]\left[ {\begin{array}{*{20}l} \mu \hfill \\ {\beta_{1} } \hfill \\ {\beta_{2} } \hfill \\ \vdots \hfill \\ {\beta_{k} } \hfill \\ \end{array} } \right]} \\ \end{array}$$

In this case, the dosages of all alleles *except one* (the reference allele) are specified. Therefore, $k$ is the number of alleles $- 1$. Each $\beta$ represents the additive genetic effect of each ancestral allele, relative to the effect of the reference ancestral.

#### Random term: kinship matrix calculation

In this model, a kinship matrix $K$ is calculated using the *realized relationship* [4]:

$$K = \frac{{DD^{t} }}{{\Delta }}\quad {\Delta } = \overline{{diag\left( {DD^{t} } \right)}}$$

where $D$ is a dosage matrix with markers on columns and individuals on rows, and the mean of each column is zero (column means have been subtracted for each column); and ${\Delta }$ is the mean of the diagonal of the $DD^{t}$ matrix. If haplotypes are used instead of biallelic SNPs, $D$ can consist of concatenated matrices similar to $X_{a}$ (without the intercept column), so that the number of columns is equal to the total number of alleles present across all markers used. To mitigate the bias due to differences in marker density across the genome, kinship estimates are calculated on a subset of evenly distributed SNPs (one marker per cM).

### Haplotyping

Haploblocks were arbitrarily defined using a sliding window of 6 consecutive SNPs with an overlap of 4 SNPs (first haplotype is SNP1-SNP2-…-SNP6, second is SNP3-SNP4…-SNP8). A haploblock of length 6 can tag a maximum of $2^{6} = 64$ alleles if all combinations are present, although in our simulations the number of observed alleles was much lower, with the average number of observed alleles ranging from 11.23 in NAM1 to 21.8 in NAM10. To obtain a haploblock position, the average position of the 6 SNP markers was taken. Haplotypes were obtained from the simulated phased SNP genotypes generated by PedigreeSim.

### Power study

#### Definition of QTL interval

Single marker QTL methods do not provide an estimate for the QTL interval, yet with a defined threshold and a genetic map one can interpret the *p* value distribution to obtain them. Since adjacent markers are not independent, and the closer to a true QTL position, the more significant the p-value becomes, one expects a chain of increasingly significant markers, pointing towards a true QTL position. Based on this, we define a QTL interval as a set of ordered markers above the significance threshold such that:

$$QTL = \left\{ {m_{1} , \ldots , m_{n} } \right\}\quad {\text{where}}\;d_{ij} < l$$

where $d_{ij}$ is the distance between adjacent significant markers $i$ and $j$, and $l$ represents a *linking distance*. As a result, a QTL interval is defined by a chain of significant markers, where adjacent significant markers are at a distance smaller than $l$. Therefore, for each value of $l$ we can define a set of detected QTL intervals. Since the choice of $l$ is arbitrary, we performed power calculations with $l$ from 0 to 10 cM in steps of 0.5 cM.

#### Significance threshold

To adjust for multiple testing, an empirical permutation threshold was calculated for each QTL analysis [20]. Thresholds were obtained with 100 permutations on a single population for each model, as threshold values did not change substantially between populations.

#### Power estimates

To evaluate the QTL models here presented we will use (1) QTL detection power, the probability of detecting a QTL position when present; (2) false positive rate, the probability of having a significant marker outside a QTL region; (3) QTL accuracy, the closeness of a QTL peak (position of maximum probability within an interval) to the true position and (4) QTL and marker precision, the probability that a significant QTL interval or marker is a true positive.

QTL detection power can be calculated as the proportion of true QTLs that are found by the model. While this is informative, one can easily increase detection power by increasing the number of false positives. To estimate the false positive rate, we must define the true negative markers (N). We considered as true negatives all markers outside a 10 cM interval around our true QTL positions (5 cM above and 5 cM below). We then define as false positives (FP) those markers that are above the significance threshold (they have lower *p* values, higher significance) and are outside the 10 cM true interval. Lastly the false positive rate is calculated as $FP/N$.

The range of a QTL interval is defined by the positions of its leftmost and rightmost markers. QTL intervals will be considered *true positives* if the QTL range includes the simulated QTL position. All markers belonging to a true positive QTL interval are considered true positive markers, whereas the rest of significant markers present in other QTL intervals will be considered *false positives*. Isolated significant markers will be ignored.

Under this framework we can define detected QTLs, true QTLs, significant markers and true positive markers. We will use these values to calculate the precision (proportion of true positives over all positives) for both QTLs and markers.

$$QTL_{precision} = \frac{true\;positive\;QTLs}{{detected\;QTLs}}$$

$$marker_{precision} = \frac{true\;positive\; markers}{{significant\; markers}}$$

Finally, we considered the ability of a model to predict the position of QTL within an interval. We can define a QTL peak as the most significant marker within a QTL interval, as is done when applying logarithm of odds (LOD) scores. QTL accuracy can then be calculated as the average distance of a QTL peak in a true QTL to the true QTL position.

Power measures were calculated for each of the three models in 11 populations for each level of genetic diversity (total of 44 populations).

### Implementation

All computations in this study were done in R [14].

Ridge regression using a restricted maximum likelihood procedure was used to obtain the mixed model estimates, which in this context are equivalent to the Best Linear Unbiased Predictions (BLUP) [21, 22]. Such calculations can be performed using the mpQTL package, where the solution algorithm, F-test approximation and *p* value calculation where based on the mixed.solve() function of the rrBLUP package [23].

To improve computational efficiency, the EMMAX/P3D approach was applied [24, 25], which approximates variance components once, and recycles these components at each marker position, reducing the amount of large matrix multiplications that must be performed.

### Simulation

#### Multiparental population design and genotype simulation

Nested Association Mapping (NAM) populations were generated using PedigreeSim V2.0, a simulation software that can simulate not only diploid but also polyploid meiosis [26]. PedigreeSim generates genotypes given a genetic map, a pedigree and the genotypes of the first generation (founders) of that pedigree. Simulations were performed using Haldane’s mapping function, allowing only bivalents with random pairing and the parameter “NATURALPAIRING” set to 1.

To speed up the calculations, an adapted tetraploid potato genetic map was used [27] containing only the first five chromosomes (3509 markers representing 485 cM). The individuals used in this study were simulated in a two-stage process: firstly, ancestor individuals were generated and used to obtain ten separate populations (ancestral groups); secondly, from each ancestral group a set of NAM founders were chosen to obtain parallel NAM populations.

For each ancestral group (AG), 10 ancestor individuals were generated with random SNP scores at each marker. Each SNP position is also given an “IBD allele”, unique to each homologue of each ancestor (even if the SNP state is the same). Each ancestral group has 10 founders, and thus a total of 40 IBD alleles will segregate in each AG. These alleles we will name *ancestral alleles.* Each ancestor is randomly crossed (without selfing, as potato is an outbreeder) to produce a first generation of 100 individuals, which will serve as parents of the second generation. This process was repeated for 50 generations, maintaining a constant generation size of 100 individuals. Finally, 100 individuals per AG were obtained as potential parents for the creation of NAM populations.

A NAM population consisted of one central parent crossed with nine peripheral parents, without any of the subsequent inbreeding that was originally proposed for NAM crossing scheme for selfing crops [12]. Each cross produced 50 offspring, thus totalling at 460 individuals per NAM. To simulate NAMs with different degrees of genetic diversity, parents were sampled from the same or from different AGs. A NAM1 contains parents from only one AG, while a NAM5 contains parents from 5 different AGs, with the same number of parents per group when possible. When the numbers of parents per AG was not equal the central parent always originated from the AG providing the most parents. For each level of genetic diversity, 11 populations were simulated. At the end of the process, the genotypes of each individual were obtained in terms of ancestral alleles (IBD alleles) and in terms of SNP dosages.

#### Phenotype simulation

Phenotypes were simulated based on the simulated genotypes: genotypic values were obtained by assigning genetic effects to the ancestral alleles at pre-defined QTL positions. Each individual will then harbour four QTL alleles at each QTL position and the final phenotype is equal to the added effects of all QTL alleles plus a normally distributed noise. No interactions between alleles in one QTL or among QTL loci were simulated, and thus additive phenotypes were obtained.

We considered a situation where three unique QTL positions (at chromosome 1, 67.88 cM; chromosome 2, 61.2 cM and chromosome 4, 100.49 cM) were segregating. Each AG has a random allelic mean, and allele effects are drawn from a normal distribution around that mean. Additionally, 50 small-effect QTLs were added randomly across the genome to simulate a polygenic effect.

For further information see Additional file 1.

## Results

### Population simulation

Ten Ancestral Groups (AGs) were simulated, each of them being founded with 40 different founder alleles. After 50 generations of random mating with a generation size of 100 individuals, each locus contained 8 to 20 founder alleles, with an average between 12.5 and 13.5 depending on the AG.

**Fig. 1:**
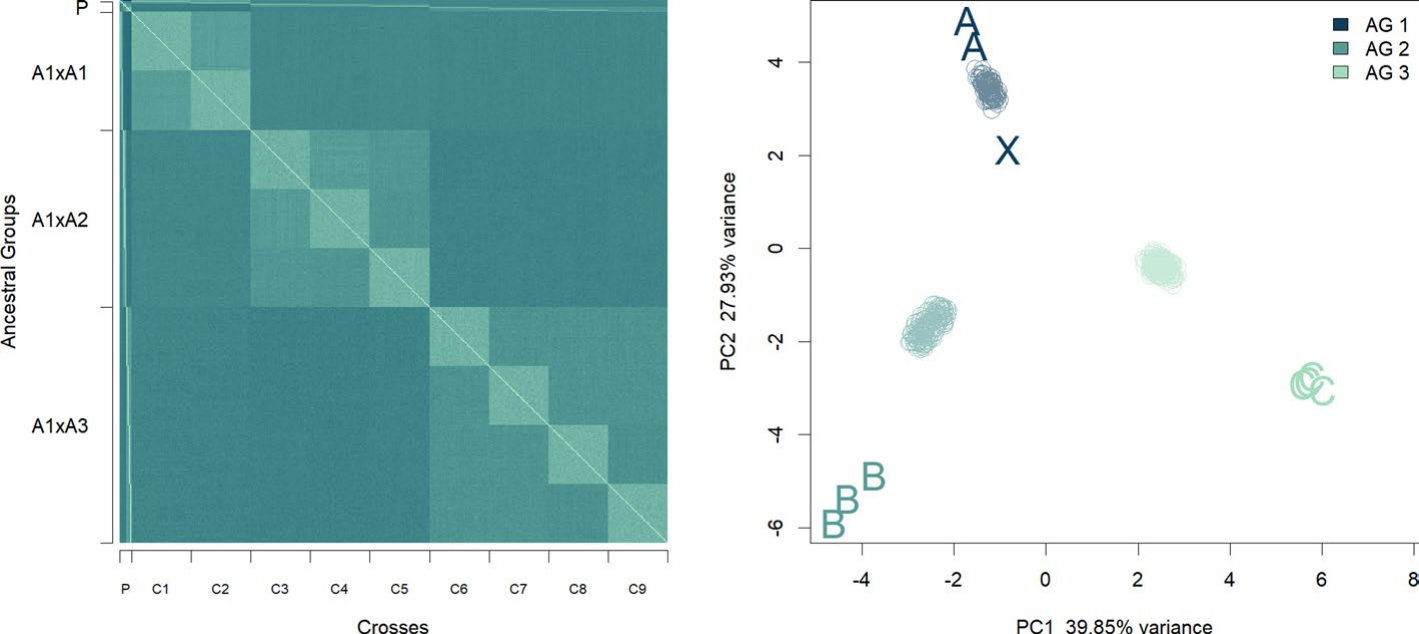
Visualization of genetic distance matrix K. Left: Heatmap of K, where lighter colours indicate higher genetic similarity between individuals. (P, parents; A*n* × Am, cross between AG *n* and AG *m*; C*n,* cross *n*). Right: Individual genotypes plotted on the two first principal components of the K matrix. Dot clouds correspond to offspring of crosses 1–9 (X, central parent, of AG1; A, peripheral parents of AG1; B, peripheral parents of AG2; C, peripheral parents of AG3)

Parents from the last generation of AGs were used to obtain NAM populations. Different degrees of genetic diversity were simulated by sampling parents from the same or different AGs, thus producing genetic structure. This is visualized for one example in Fig. 1, which shows a heatmap of the relatedness matrix $K$ and a Principal Coordinate Analysis (PCoA) plot of the same matrix. On the left, we see how cross 3, 4 and 5, derived from crosses between AG1 and AG2 (A1 × A2 in Fig. 1), have a higher relatedness between them than with any other cross. Similarly, in the PCoA plot we observe how the individuals from these crosses (light blue dot cloud) cluster together in the midpoint between X (from AG1) and the three parents B (from AG2). These indications confirm that our two-step approach was successful in generating NAM populations with genetic structure. A similar outcome can be observed in the NAM1 to NAM10 simulations.

### Population comparison

**Fig. 2:**
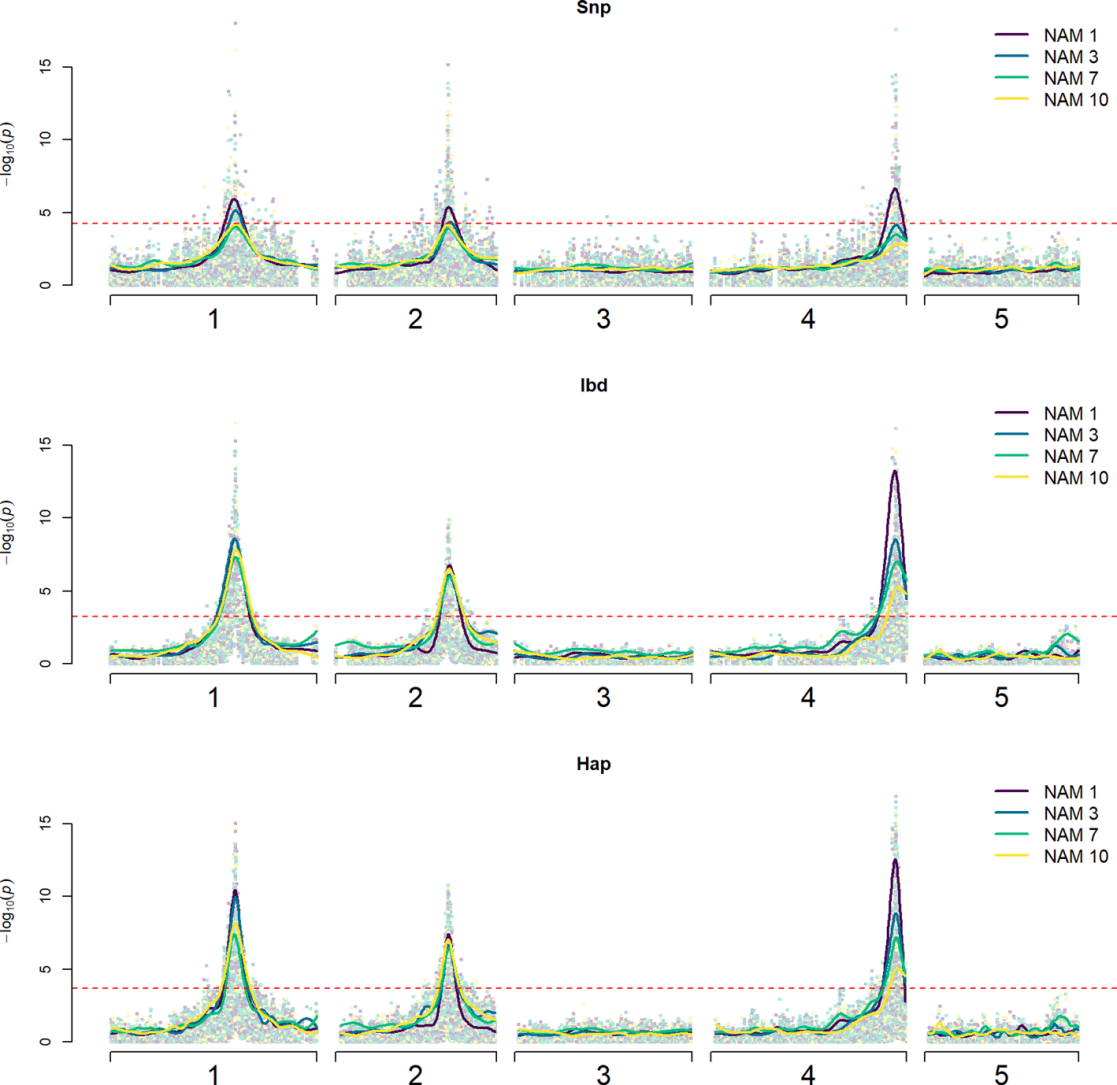
Overlap of *p* value distribution across all populations in the three models. Top, biallelic SNP model; middle, multiallelic IBD model; bottom, multiallelic haplotype model. Coloured solid lines represent the 99th percentile of all *p* values observed in each genetic diversity level at a particular position. The red dotted line marks the estimated permutation threshold for each model (SNP: $10^{ - 4.22}$, IBD: $10^{ - 3.27}$, haplotype: $10^{ - 3.67}$)

For each level of diversity, 11 populations were tested with the three proposed models. In almost all cases, al models were able to detect all QTL regions. Regardless of the linking distance used for QTL estimation, lower diversity resulted in higher detection power (Table 1). This can be observed at $l = 3$ using haplotype markers: NAM1 has a detection power of 1 (all QTLs were found in the 11 populations), but this power decreases to 0.818 in NAM10. Similarly, the false positive rate decreases as diversity increases and is lowest in the SNP model than in IBD or haplotype models. In Fig. 2 the 99th percentile profiles also highlight the increased power in lower diversity populations, where the dark blue line representing NAM1 populations had higher significance values for all QTL peaks and for all models. As diversity increases, a similar decrease can be observed for QTL precision. Finally, the mean peak distance from the QTL peak to the true QTL position was also larger (lower accuracy) at a higher level of diversity in the populations (Table 1).

**Table 1:** Power comparison across genetic diversity and marker types. Each estimate is an average of 11 populations for each diversity level, with $l = 3 \;{\text{cM}}$. SNP refers to the biallelic model, IBD refers to the ancestral, multiallelic model and Hap refers to the haplotype-based approach. For detection power and QTL precision, higher numbers indicate a better model, while for false positive rate and accuracy, lower numbers indicate a better model

	Detection power			False positive rate			QTL precision			Accuracy (cM from true position)		
	SNP	IBD	Hap	SNP	IBD	Hap	SNP	IBD	Hap	SNP	IBD	Hap
NAM1	0.939	1	1	0.012	0.066	0.055	0.917	0.850	0.941	0.593	0.161	0.121
NAM3	0.909	0.970	0.970	0.008	0.065	0.054	0.865	0.814	0.886	0.550	0.192	0.130
NAM7	0.545	0.939	0.909	0.005	0.064	0.040	0.697	0.842	0.879	0.687	0.325	0.331
NAM10	0.606	0.848	0.818	0.005	0.055	0.038	0.773	0.932	0.850	0.665	0.312	0.621

### Marker comparison

**Fig. 3:**
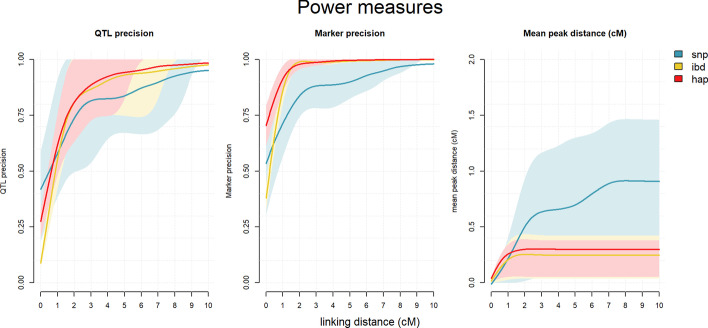
Power measures for each model with different values of *l*. Power was calculated with $l = 0 \;{\text{to}}\;10$ in steps of 0.5 cM over 44 NAM populations (11 of each: NAM1, NAM3, NAM7 and NAM10), for the SNP dosage model (snp), true IBD model (ibd) and haplotype model (hap). Coloured areas represent the 20th to 80th percentile of power values for each model, and trend lines represent the average of each. Both lines and area edges where smoothed using a LOESS regression

**Fig. 4:**
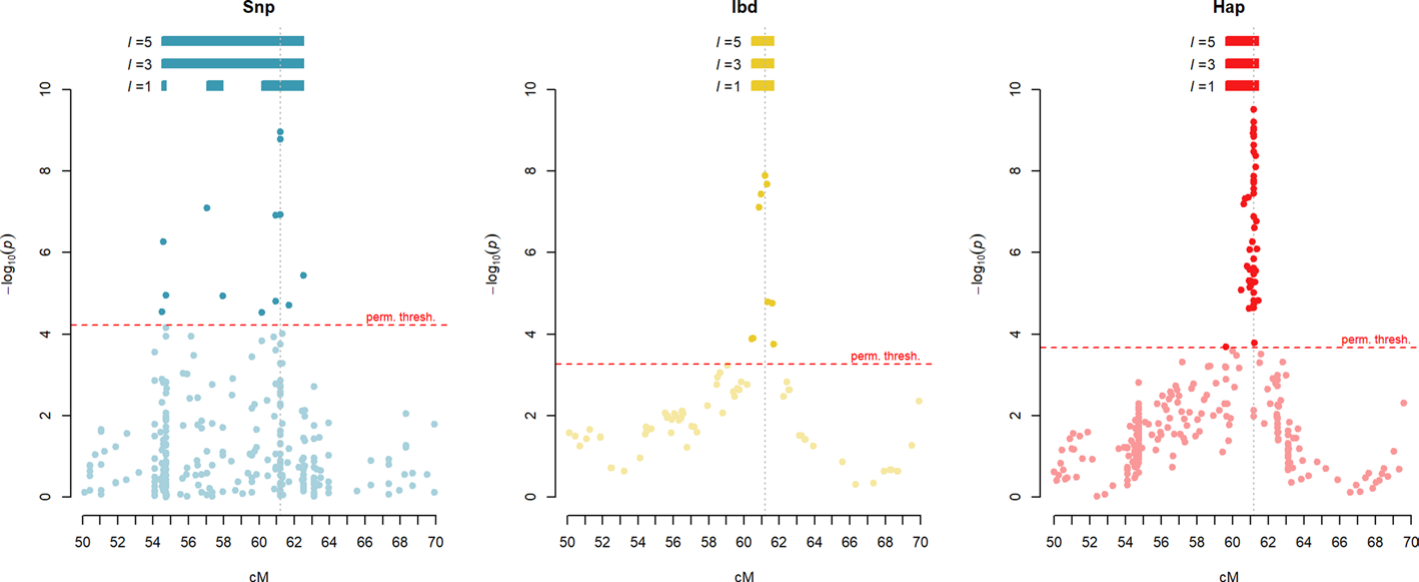
Example of QTL interval detection. *p* value distributions are shown for the same region in the same population using the three models (left, SNP; middle, IBD and right, haplotype). Detected QTLs are presented above each plot for three values of *l*: 1, 3 and 5 cM. Horizontal red dotted lines represent the permutation threshold for each model, and grey vertical lines highlight the true QTL position. In the SNP model with *l* = 1 cM, three QTL intervals are detected, of which only one contains the true QTL position, while with higher values of *l,* only one QTL interval is detected. In the IBD and haplotype models, a single true QTL is detected for all *l* values shown

Across NAM populations and at a linking distance ($l$) of 3 cM, detection power averaged at 0.74 for SNPs, 0.93 for IBD and 0.92 for haplotypes and was stable for $l > 1$ cM. The decrease in detection power as genetic diversity increased was markedly larger in the SNP models than in the multiallelic models (Table 1). This can be clearly observed in the 99^th^ percentile lines in Fig. 2: when diversity increases, the trend line is below the significance threshold in the SNP models, while for both multiallelic models all trend lines stay well above their respective thresholds. In Fig. 3 left and centre panels, we can see how the proportion of true positives increases as the value of *l* increases. For $l > 1$ cM, QTL precision is on average higher for multiallelic models (0.91 IBD, 0.92 haplotype) than for the SNP model (0.86). Marker precision is also higher for the multiallelic models (0.99 IBD, 0.99 haplotype, 0.92 SNP). The choice of $l$ has an impact on this difference, as for lower values of $l$ (but above 1) precision is much lower for the SNP model. This is due to the presence of significant markers further away from the true QTL position in the SNP model than in the multiallelic models (Fig. 4).

Peak accuracy (Fig. 3, right panel) is stable from $l > 1$ at 0.25 cM for IBD and 0.30 cM for haplotype models. In the SNP model, peak accuracy is lower and shows more variation. At $l = 1$ peak accuracy is similar to the IBD and haplotype models, yet many false positives are present in the QTL analysis (see Fig. 4). At higher $l$, average peak distance increases from 0.33 cM at $l = 2$ to 0.83 cM at $l = 7$.

## Discussion

### Model comparison

The essence of a QTL study is the genetic linkage between observed markers and unobserved QTL alleles. When dense genetic maps are used, the purpose of a QTL model should be to obtain an increasing marker significance as the analysis approaches a true QTL position. The definition of QTL interval used in this study stems from such reasoning: we expect a chain of contiguous significant markers that form a peak structure, pointing towards the true QTL position.

Classical QTL experiments were carried out on inbred diploid experimental crosses. In this setup one can expect only two alleles per QTL to segregate, and thus biallelic SNP markers are able to uniquely tag each allele. In this context, a SNP regression is equivalent to testing the difference in phenotype due to having 0, 1 or 2 copies of each marker allele [1, 28]. However, when we move to scenarios where more than two alleles per QTL are expected to segregate at a single locus, for instance when heterozygosity is expected to be high or in multiparental populations, single SNPs no longer tag QTL alleles uniquely. Thus, each SNP allele might tag more than one functional QTL allele, creating a situation where the regression test is being performed between groups that do not represent a unique effect. Only if, by chance, those groups happen to divide functional alleles between those with large effects and those with small effects, will SNP markers be significant. Since two factors are affecting the significance of biallelic markers (i.e. distance to the true QTL position and the grouping of multiple effects), they become worse at estimating the true QTL position.

Figure 4 illustrates this situation. The three panels represent the same population being analysed with the three models presented in this study. It can be seen how in the SNP model there are three significant markers at approximately 54.5 cM, while the true QTL position is at 61.2 cM. Meanwhile there are quite some markers near the true position that are not significant. Such behaviour is not seen in the multiallelic models where markers near the true QTL position form a clear peak and more distant markers show no significance.

The consequences of this can be seen in Figs. 2 and 3. First, SNP models have overall lower significance at the QTL regions (Fig. 2), an effect that is increased when genetic diversity increases and biallelic markers become increasingly worse at tracking the multiple effects present in the population. This explains the lower detection power of biallelic models when genetic diversity is increased (Table 1). Secondly, we see how at low linking distances, SNP models have a high number of significant markers in false-positive QTL intervals (Fig. 3 middle). As *l* is increased, marker precision increases (there are less false-positive QTLs), but at the cost of accuracy (Fig. 3 left): the QTL intervals become larger (Fig. 4), including markers at some distance of the QTL position with higher significance than those at the simulated QTL position.

Thus, in a context of high genetic diversity, the usefulness of SNP models will depend on marker density, as higher density gives higher chances of having at least one marker at the QTL position that divides functional QTL alleles in two groups with statistically different means. Even if such a marker is found and the location of the QTL is detected, the effect estimated by a regression model does not realistically represent the true functional alleles present in the population.

Considering the lower detection power, lower accuracy and inability of biallelic QTL models to estimate effects for multiple alleles, it is clear that SNP-based biallelic models are a limited and limiting tool when applied to multiallelic populations.

### Multiallelic markers

In order to apply multiallelic models, one must be able to obtain multiallelic genotypes. One possibility is to utilize markers that are multiallelic per se, such as SSR markers, but these markers are less common along the genome, their detection cannot be automated, and they are therefore hard to apply within high-throughput pipelines.

Alternatively, several studies have proposed the use of multiallelic haplotypes: groups of phased adjacent SNPs. This type of markers has the advantage of being predictive of two parts of IBD: family IBD, regions of chromosomes from two individuals that originate from the same *parental chromosome*; and ancestral IBD, chromosomal regions originating from the same *ancestral chromosome* that could occur in more than a single founder and that are broken down by recombination events [29].

While in our simulations haplotyping was trivial because the genotype of each individual was known, haplotyping of real SNP data requires *phasing*. For instance, if two adjacent marker genotypes of an individual are *AAAB* and *AAAB*, the underlying four haplotypes could be both *AA*-*AA-AB-BA* or *AA-AA-AA-BB*. Some approaches have been developed for haplotyping in polyploids [30–32] but regardless of the method, haplotype estimation from SNP data carries a certain degree of uncertainty due to the high number of possible solutions with similar probabilities. This uncertainty is not present in the haplotypes used in this study, meaning that the haplotype model here presented is performing better than what would be expected with real data, depending on the accuracy of haplotype estimation.

Nevertheless, sequencing technologies are becoming a mainstream approach for genotyping, and haplotypes can be directly observed in longer sequencing reads. Identifying haplotypes for different individuals given a set of reads is a complex mathematical problem that has spurred the development of a variety of tools [31, 33–35]. The haplotypes obtained from these methods could also be used with the multiallelic polyploid model introduced in this paper, allowing to perform QTL analysis in genetically diverse polyploid populations based on sequence data.

Lastly, in this simulated population each founder allele had a different QTL effect. In nature this might not be the case, as it is well known that many mutations are in fact neutral and thus do not change the QTL effect of that mutated allele. This could imply that the number of haplotypes would be higher than the number of QTL effects in a population, thus decreasing the usefulness of haplotype-based multiallelic markers.

### Preparing multiparental populations

When organizing an MPP, the power to be able to detect the effects of an allele at a QTL depends on its frequency. The more individuals harbour one QTL allele, the more information the MPP provides about it. The expected frequency of founder alleles is directly affected by two factors: founder genetic diversity and offspring per founder.

The number of alleles segregating in a population is a direct reflection of the genetic diversity of its founders. When relatedness between founders is high, the chances of two founder chromosomes harbouring the same allele is also high. In MPPs where founders are very related, ultimately not many alleles can be expected to segregate. In contrast, when relatedness between founders is low, they have high chances to contribute unique alleles. The approach here presented estimates one parameter per each allele in the population, and thus, if population size is maintained constant, the power of the model decreases as the number of alleles increases. This hypothesis was confirmed by our simulation study where systematically, higher diversity populations, which require more allele effect parameters, presented lower QTL detection power, lower precision and lower QTL accuracy (Fig. 2, Table 1).

A second aspect to be considered is the number of offspring per founder. The larger the contribution of a founder to the individuals of the MPP, the higher the power to detect and estimate the effects of its alleles [36]. For instance, using our NAM design, the alleles present in the central parent were present in all crosses. Alleles from peripheral parents not shared with the central parent had fewer individuals contributing to their effect estimation, meaning these estimations will be less powerful.

Considering the previous points, we suggest that MPPs should be developed with an intermediate diversity and ensuring that those alleles to be studied are kept at a relatively high frequency. Following this logic, a few parents from the same ancestral group (AG) can be selected (which likely share some alleles) and crossed with several other AGs. If all AGs are equally interesting for the QTL study, then all AGs should have a similar contribution to the offspring [36]. If an MPP is designed from an already-existing set of connected F1 crosses, then each cross should be of similar size and the number of crosses per AG should be similar. When more complex pedigrees are used, ancestry coefficients can help guide the design of MPP.

## Conclusion

Genetic diversity is the basis of breeding, and thus, characterizing it becomes essential in the development of new varieties. The methods developed within the “mpQTL” package add to the growing toolset for polyploid organisms. It is now possible to apply multiallelic models in polyploid organisms in the presence of genetic structure, which we have shown are more powerful, especially in the presence of high genetic diversity. Additionally, this study supports an alternative approach to the study of genetic diversity. Instead of using a diversity panel to perform a GWAS, a selection of these diverse accessions can be used as founders of an MPP. Each biparental cross within the MPP will add information to the QTL study, and future crosses can be added to the overall MPP analysis. This approach shows much promise in the context of breeding, particularly for its ability to connect and share information between crosses that in traditional approaches would remain separate.

## Supplementary Information

**Additional file 1**. Allelic effect sampling and phenotype simulations.

## Data Availability

The genotypes and phenotypes generated and analysed during the current study are available in the Figshare repository: 10.6084/m9.figshare.14315867. The QTL results generated and analysed during the current study are available in the Fighsare repository: QTL results, 10.6084/m9.figshare.14316068.

## Code availability

PedigreeSim can be found on GitHub: https://github.com/PBR/pedigreeSim.

The package mpQTL together with vignette and example datasets can be found on Github (https://github.com/Alethere/mpQTL).

The code for producing the datasets used in this article can be found on Figshare: 10.6084/m9.figshare.14316107.
