# Supplementary material for: Multiallelic models for QTL mapping in diverse polyploid populations
Source: BMC Bioinformatics. 2022 Feb 14;23:67. doi: 10.1186/s12859-022-04607-z (PMC8842866; doi:10.1186/s12859-022-04607-z)
Supplement: Supplementary file 1 — Additional file 1. Allelic effect sampling and phenotype simulations. [file 12859_2022_4607_MOESM1_ESM.pdf]

Supplementary information

GENETIC MODEL SELECTION AND PHENOTYPE SIMULATION

Simulating phenotypes is a challenging process as countless genetic situations might be generated: one big QTL effect and several small ones, many randomly sized QTLs, etc. An educated choice must be taken considering the type of research question to be addressed.

In this study, our objective was to characterize the statistical power of the IBD model in multiparental populations of polyploids. Ancestral groups represent closed populations from which parents are sampled and were created to simulate different degrees of genetic similarity between parents. This approach emulates common scenarios in plant breeding, where diversity is structured in gene pools due to geographic isolation and differential selection pressures between pools (e.g. Balfourier *et al.* 2018).

Genetic model

To describe our phenotypic simulation methods, let us consider a phenotype that is defined by:

$$y_i = G_i + E_i \quad E_i \sim N(0, \sigma_E^2)$$

Where y_i is the phenotype of individual i, with genetic effect G_i and random residual effects E_i, distributed as a normal random variable with mean 0 and variance σ_E^2. For simplicity, we simulated **additive QTLs** with no inter QTL interactions. Following the notation presented in Materials and Methods, the genetic effect can be defined as the sum of the allele effects multiplied by the dosage of each allele. Thus, for a phenotype defined by QTLs $l = \{1, \ldots, L\}$, each QTL containing $j = \{1, \ldots, k_l\}$ alleles and each allele having an effect α_{jl} (for the size of genetic effects, see following section), we can express the value y_i as:

$$y_i = \sum_{l=1}^{L} \left(\sum_{j=1}^{k_l} \delta_{ijl} * \alpha_{jl} \right) + E_i$$

As heritability is a relevant parameter in QTL analysis, we might be interested in controlling it. Let us define a general heritability (of all QTLs combined) as $h^2 = \sigma_A^2 / (\sigma_A^2 + \sigma_E^2)$, where σ_A^2 is the additive variance (in our case also the total genetic variance, as all genetic effects are additive). Assuming independence between genetic and environmental effects, we can rescale the genetic effects to achieve a heritability h^{2*} using the following formula:

$$\alpha_{jl}^* = c\alpha_{jl} \qquad c = \sqrt{\frac{h^{2*}\sigma_E^2}{(1 - h^{2*})\sigma_A^2}}$$

Where α_{jl}^* represents the rescaled genetic effects. Additionally, a "polygenic term" has been added to the phenotypes, which is intended to increase family phenotypic

resemblance. The polygenic term was generated by selecting 50 random positions along the genome, and assigning genetic effects distributed normally following $e_i \sim (\mu = 0, \sigma = 0.1)$ where e_i corresponds to the genetic effect on locus i and 0.1 is the standard deviation of the normal distribution. These genetic effects were assigned without taking into account the AG of the alleles, thus simulating family-relatedness rather than ancestral relatedness. As a result, genetic effects on polygenic loci ranged from -0.37 to 0.32, with an average of 0.002.

Choice of genetic effects

The number of genetic effects that must be obtained will depend on the number of different ancestral alleles present at each position, and thus is both position and population dependent. In a NAM3 population we have on average 11 alleles per locus. We simulated genetic effects in such a way that each ancestral group contributed functional alleles (alleles with a nonzero effect) for three QTLs positions. Once these alleles have been assigned we must assign allele effects. To do so, we considered three different scenarios:

1) Allelic effects within ancestral groups are very similar, but very different between ancestral groups. Those are genes that are very strongly selected (a trait that is essential for survival) but with a different effect (a different phenotypic value) being selected in each ancestral group.
2) Allelic effects with some variation within ancestral groups, but also some variation between ancestral groups. We could imagine a gene contributing to a non-essential trait of which multiple variants exist in each ancestral group, but that have different means for each ancestral group.
3) Allelic effects where the ancestral group has no influence on the effect distribution. Highly variable genes under diversifying selection would behave in such manner.

Once a scenario has been chosen, the allelic effect choice was performed as follows:

1. An ancestral mean μ_{AG} is chosen randomly from a uniform distribution. Since the effects will be scaled when the heritability control is performed, only the relative size between means is relevant.
2. Genetic effects are chosen from a normal distribution so that $\alpha \sim N(\mu_{AG}, \sigma^2)$, where we consider all ancestral groups to have the same variance σ^2.

When σ^2 is much smaller than the difference between the μ_{AG}, we will simulate scenario 1. On the other extreme we find scenario 3, where the size of σ^2 is so large that differences between μ_{AG} are not meaningful. It is in the intermediate point between these two extremes where we can find scenario 2. Since it was not evident which scenario would be more interesting for our study, we simulated high heritability phenotypes with all of them and applied the ancestral model with true IBD alleles (Fig. S1)

We observed that in scenario 1, no peaks were found back, while scenario 3 had the strongest peaks and scenario 2 still detected peaks but of smaller size. Scenario 1, where

all genetic diversity is *between* ancestral groups, rather than *within* ancestral groups has no detection power due to the structure correction we perform. Since we are eliminating structure-associated phenotype variation (in scenario 1 this is in fact all variation) the only phenotypic variation left is noise, and thus no QTL peaks are detected. While scenario 3 offers the most power, it seems unlikely that there would be no relationship between ancestral group and genetic effect. Thus, **we performed simulations using scenario 2.** In practice, that meant generating phenotypes using three random means between 0 and 1 with $\sigma^2 = 1$.

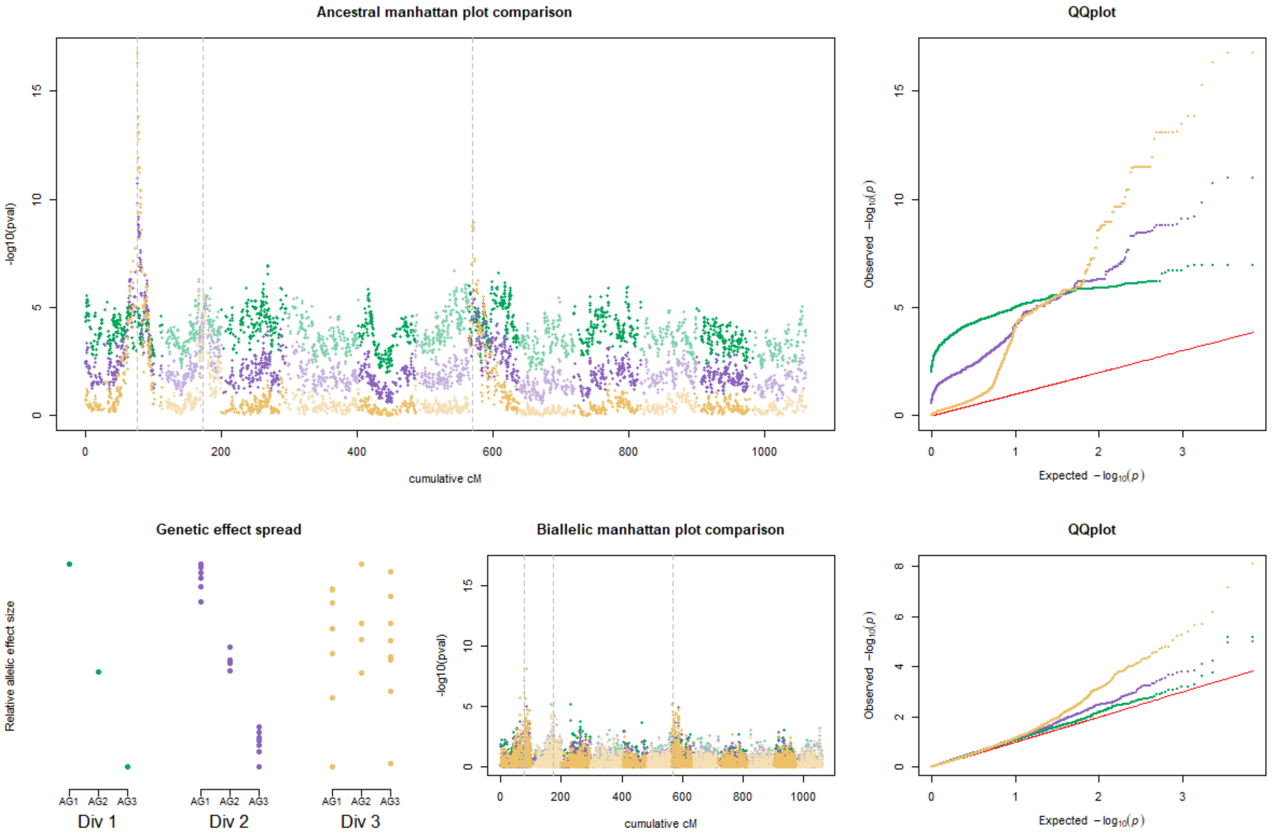

Figure S1: Genetic effect diversity scenarios. When there is a high correlation between phenotype and genetic structure, that is, when certain effects are present only in subsets of the population, the models become insensitive. In green we see a case where all the effect variance is present between AGs (Div 1), in purple when variance is divided between and within AGs (Div 2) and in ochre, the case when effect variance is only present within AGs and all AGs harbour similar effects (Div 3). **Top left:** overlap of three effect models for an ancestral model. The diversity scenario 1 is the least powerful since the structure correction is elimination all population variation, in scenario 2 there is some detection although p-values remain inflated and in scenario 3 there is no inflation and QTL peaks are clearly detected. **Top right:** QQ-plot of the p-values in the top left panel, highlighting the p-value inflations seen in scenarios 2 and 3. **Bottom left:** graphical representation of the relative effect sizes for alleles of each AG in each of the diversity scenarios. **Bottom middle:** same Manhattan plot as in top left but using a biallelic model. In this case there is no inflation since alleles are not nested within certain parts of the population. **Bottom right:** QQ-plot of the bottom middle panel. We see how in this case there is no inflation.
